# Transition-Metal Sulfide/Carbon Composites Derived from Recycled Sulfonated Resin as High-Photothermal-Conversion Materials for Solar-Driven Interfacial Evaporation

**DOI:** 10.3390/ma19194238

**Published:** 2026-10-06

**Authors:** Gexin Zhao, Meiyan Guo, Ao Liu, Jianming Tang, Gang Xiong, Guangwen Xu

**Affiliations:** 1 School of Chemistry and Chemical Engineering, Liaoning University of Science and Technology, Anshan 114051, China; 2 Key Laboratory on Resources Chemicals and Materials of Ministry of Education, Shenyang University of Chemical Technology, Shenyang 110142, China

**Keywords:** Amberlyst 15 resin, transition-metal sulfide, photothermal conversion, interfacial water evaporation

## Abstract

The objective of this study is to investigate the recycling and utilization of Amberlyst 15 resin, which contains sulfonic acid groups, with the aim of transforming it into an excellent photothermal conversion material to enhance interfacial water evaporation capability. By pyrolyzing the resin and transition metal salt composite, composite materials of transition metal sulfides and carbon (Cu_7_S_4_-C, Ni_3_S_2_-C, and Co_3_S_4_-C) were successfully prepared, which combine the electron-phonon coupling effect and localized surface plasmon resonance (LSPR) effect. The prepared materials were characterized in detail. The results of evaporation experiments using distilled water indicated that the net evaporation rates of water evaporators constructed from Cu_7_S_4_-C, Ni_3_S_2_-C, and Co_3_S_4_-C reached 1.23, 1.06, and 1.15 kg·m^−2^·h^−1^, respectively, under one-sun irradiation, exhibiting photothermal conversion efficiencies of 85.7%, 73.8%, and 80.1%, respectively. This study proposes a sustainable resource utilization method for solid waste sulfonic acid resin, and based on this, develops a new type of photothermal conversion material suitable for solar-driven interfacial water evaporation systems.

## 1. Introduction

With the rapid development of modern industry and the continuous growth of the global population, the issues of water pollution and water scarcity have become increasingly severe, garnering widespread attention from countries around the world [1,2]. Traditional water desalination technologies such as reverse osmosis [3] and thermal desalination [4] have revealed shortcomings in commercial development and application, including high manufacturing costs and significant resource consumption [5]. In recent years, numerous studies have shown that solar interfacial evaporation technology is an emerging, highly efficient solar utilization technology with great potential, offering advantages such as high evaporation efficiency, low energy consumption, environmentally friendly processes, and sustainability [6,7]. In this technology, photothermal conversion materials play a pivotal role in determining the utilization efficiency of solar energy. Currently, various types of photothermal conversion materials such as semiconductor materials [8], carbon-based materials [9], metal materials [10], and polymer materials [11] have been reported. Carbon-based materials have become one of the most efficient and stable photothermal conversion materials due to their wide-spectrum absorption, excellent thermal conductivity, and tunable microstructure [9]. Nevertheless, researchers are striving to enhance their photothermal conversion efficiency through two strategies: firstly, porous carbon materials are constructed to enhance light adsorption, such as carbon gel [9,11], and secondly, the materials are combined with substances capable of generating the LSPR effect, such as gold and silver nanoparticles [12,13].

Amberlyst 15 resin is a representative of macroporous strongly acidic resin, widely used as an acid catalyst in heterogeneous systems [14,15]. However, in the event of a failure in its catalytic function or excessive swelling, the material will transition into a state of solid waste. The prevailing treatment method entails the incineration of the material, a process that has been shown to result in significant resource wastage [16]. Amberlyst 15 resin contains significant quantities of carbon and sulfur elements. The conversion of these elements into functional materials during the carbonization process would be a significant development. In this study, Amberlyst 15 resin was pyrolyzed in the presence of transition metal nitrates (copper, nickel, and cobalt). The sulfur element in Amberlyst 15 resin was then reacted in situ with the metal ions to prepare sulfides, thereby preparing functional transition metal sulfide/carbon composites (Cu_7_S_4_-C, Ni_3_S_2_-C, and Co_3_S_4_-C). A comprehensive characterization of transition metal sulfide/carbon composites was conducted, and the constructed evaporators were utilized to assess their water evaporation performance.

## 2. Experimental Section

### 2.1. Materials and Physical Measurements

All reagents for the syntheses were of analytical grade and were used as received from commercial sources without further purification. Amberlyst 15 resin was purchased from Alfa (Ward Hill, MA, USA) and used for Wagner-Meerwein rearrangement of alcohols before being used in this study. Elemental analysis (CHNS) was performed using an Elementar UNICUBE organic elemental analyzer (EA; Elementar, Hanau, Germany). FTIR spectra were recorded on a Nicolet 6700 spectrometer (Thermo Fisher Scientific, Waltham, MA, USA). Metal concentrations in water samples were determined by an iCAP TQ inductively coupled plasma mass spectrometer (ICP-MS; Thermo Fisher Scientific, Waltham, MA, USA). The scanning electron microscopy (SEM) images were recorded using JEOL-2100F and JSM-4800F electron microscopes (JEOL, Tokyo, Japan). Powder X-ray diffraction tests were performed using a D8 ADVANCE polycrystalline X-ray diffractometer (Bruker, Karlsruhe, Germany) at room temperature. UV-Vis-NIR spectra were measured using an Agilent Cary 5000 spectrophotometer (Agilent, Santa Clara, CA, USA). X-ray photoelectron spectroscopy was performed using a Model Axis Supra+ (Shimadzu/Kratos, Kyoto, Japan). The photothermal conversion efficiency of the materials was tested using a PLS-SXE300 xenon lamp from Perfectlight^®^ (Beijing, China) to simulate sunlight. A light intensity meter (CEL-NP2000) was purchased from Beijing China Education Au-light Co., Ltd. (Beijing, China).

### 2.2. Preparation of the Composite Materials

**Figure 1:**
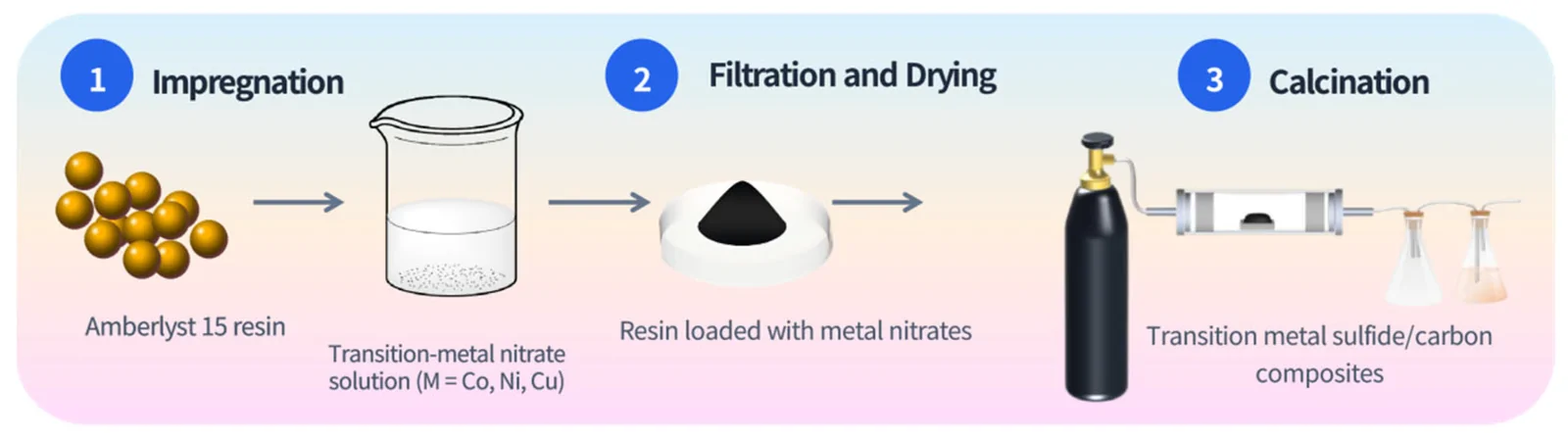
Schematic illustration of the synthesis of Cu_7_S_4_-C, Ni_3_S_2_-C, and Co_3_S_4_-C.

As shown in Figure 1, Amberlyst 15 resin was immersed in aqueous solutions containing an equal weight of cobalt nitrate hydrate for 8 h. After complete impregnation, the mixture was filtered, and the filter cake was dried in an oven at 90 °C to obtain a powdered precursor. Then, the dried precursor (8.0 g per batch) was placed in a tube furnace and heated under an argon atmosphere (about 50 mL·min^−1^). The temperature was raised from room temperature to 800 °C in 1 h and maintained at 800 °C for 2 h. The black powder Co_3_S_4_-C material was obtained. Then, cobalt nitrate hydrate was replaced with nickel nitrate hydrate and copper nitrate hydrate, respectively, and according to the aforementioned experimental conditions and steps, Ni_3_S_2_-C and Cu_7_S_4_-C materials were prepared, respectively. For comparison, a metal-free carbonized Amberlyst 15 sample (denoted as C-Amberlyst) was prepared under identical conditions but without immersion in any metal nitrate solution.

### 2.3. Interface Evaporation Experiment

The indoor environment for the light-driven evaporation experiment conducted in the laboratory had a temperature of around 25 °C, a water temperature of around 25 °C, and an ambient humidity not exceeding 50%. A 20 mg amount of transition-metal sulfide/carbon composites was mixed evenly in polyvinyl alcohol, and the slurry was applied to the surface of a square lignocellulosic sponge (3 cm × 3 cm × 0.2 cm) using a paintbrush, followed by drying at 65 °C for 30 min to evaporate excess water. Each evaporation test lasted 60 min. All measurements were repeated three times, and the average values with standard deviations were reported. In the solar-driven evaporation experiment, expandable polystyrene (EPS) foam was used as both a self-floating material and a thermal insulation layer. The EPS foam was wrapped in cotton cloth, which served as a water transport material. The wet sample was placed on the cotton cloth, and the remaining part was covered with 0.5 mm EPS foam. The aforementioned materials were placed in a box containing 50 g of water. For the long-term stability and leaching tests (336 h), the water volume was increased to 1000 mL. A xenon lamp with a light intensity of 1.0 kW·m^−2^ was used as the light source, and an electronic balance with an accuracy of 0.1 mg connected to a computer was used to record the mass change during evaporation in real time. The surface temperature of the evaporator under illumination was monitored using an infrared camera. The light intensity was calibrated using a light intensity meter.

## 3. Results and Discussion

### 3.1. Characterization of Photo-Thermal Conversion Materials

Elemental analysis (CHNS) was performed to determine the composition of the dried Amberlyst 15 resin, and the results are summarized in Table S1. The resin contained C 43.88 wt%, H 5.35 wt%, S 18.26 wt%, and O 32.51 wt%. This composition confirms that the resin can serve as both a carbon and sulfur source for the in situ formation of transition-metal sulfides during pyrolysis. The thermal decomposition behavior of the Amberlyst 15 resin was investigated using thermogravimetric analysis (TGA) and derivative thermogravimetry (DTG), and the results are presented in Figure S1. Thermogravimetric analysis (TGA-DTG) results show that the Amberlyst 15 resin exhibited a total weight loss of 59.47% from 30 to 797 °C, with a final residue of 40.56%. Its thermal decomposition occurred mainly between 200 and 330 °C, corresponding to the decomposition of sulfonic acid groups and the initial cleavage of the polystyrene-divinylbenzene backbone, along with the release of sulfur-containing species that can react in situ with transition-metal ions to form metal sulfides. Above 330 °C, further degradation of the polymer framework and progressive carbonization/condensation of the carbon residue took place. These results confirm that the 800 °C pyrolysis treatment is sufficient to decompose the sulfonic acid groups, release sulfur for in situ sulfidation, and carbonize the polymer matrix. Moreover, the high residual mass indicates that the resin can serve as an effective carbon precursor and support for the preparation of Cu_7_S_4_-C, Ni_3_S_2_-C, and Co_3_S_4_-C photothermal materials.

**Figure 2:**
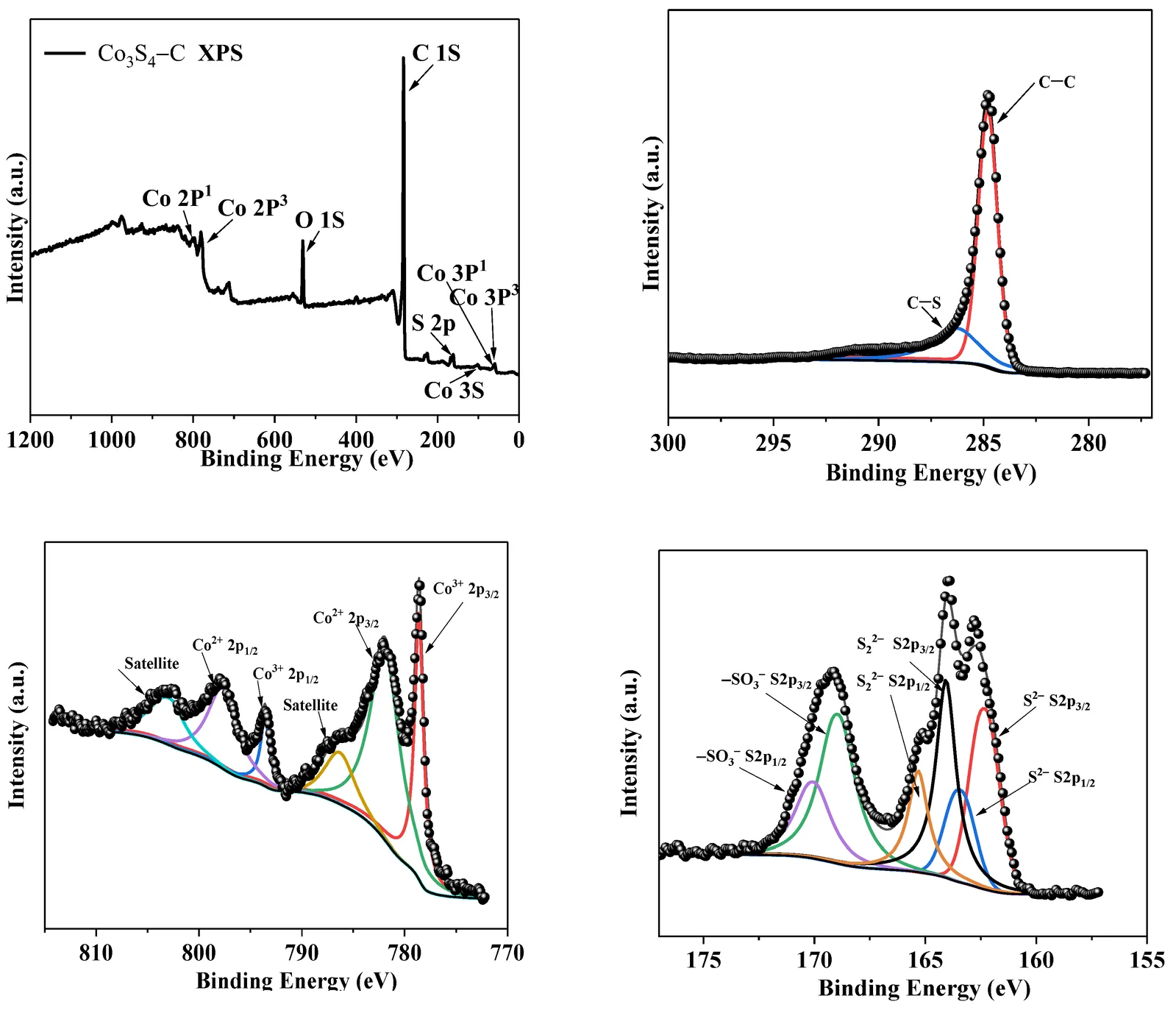
XPS spectra of Co_3_S_4_-C material.

**Figure 3:**
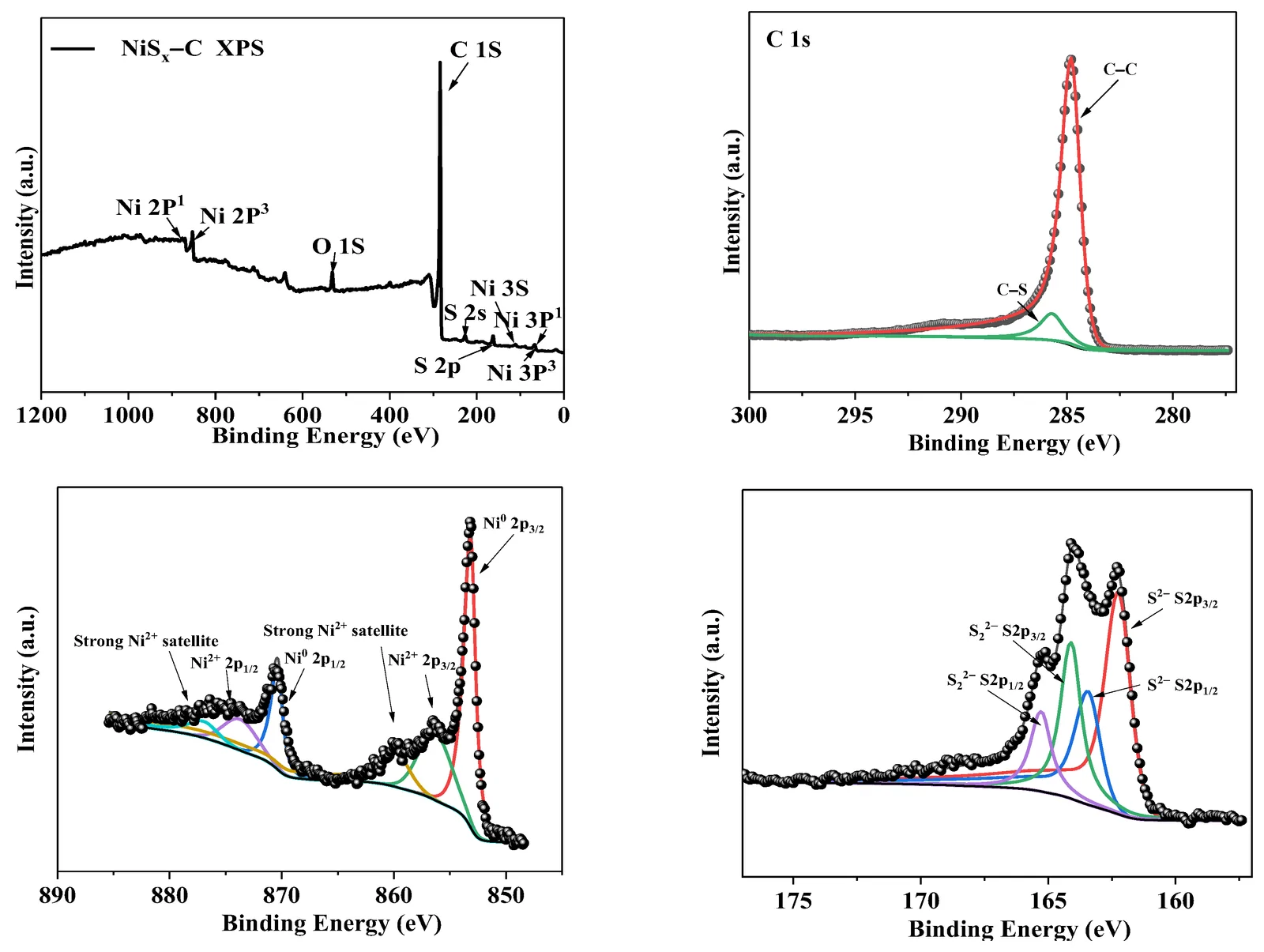
XPS spectra of Ni_3_S_2_-C composite.

**Figure 4:**
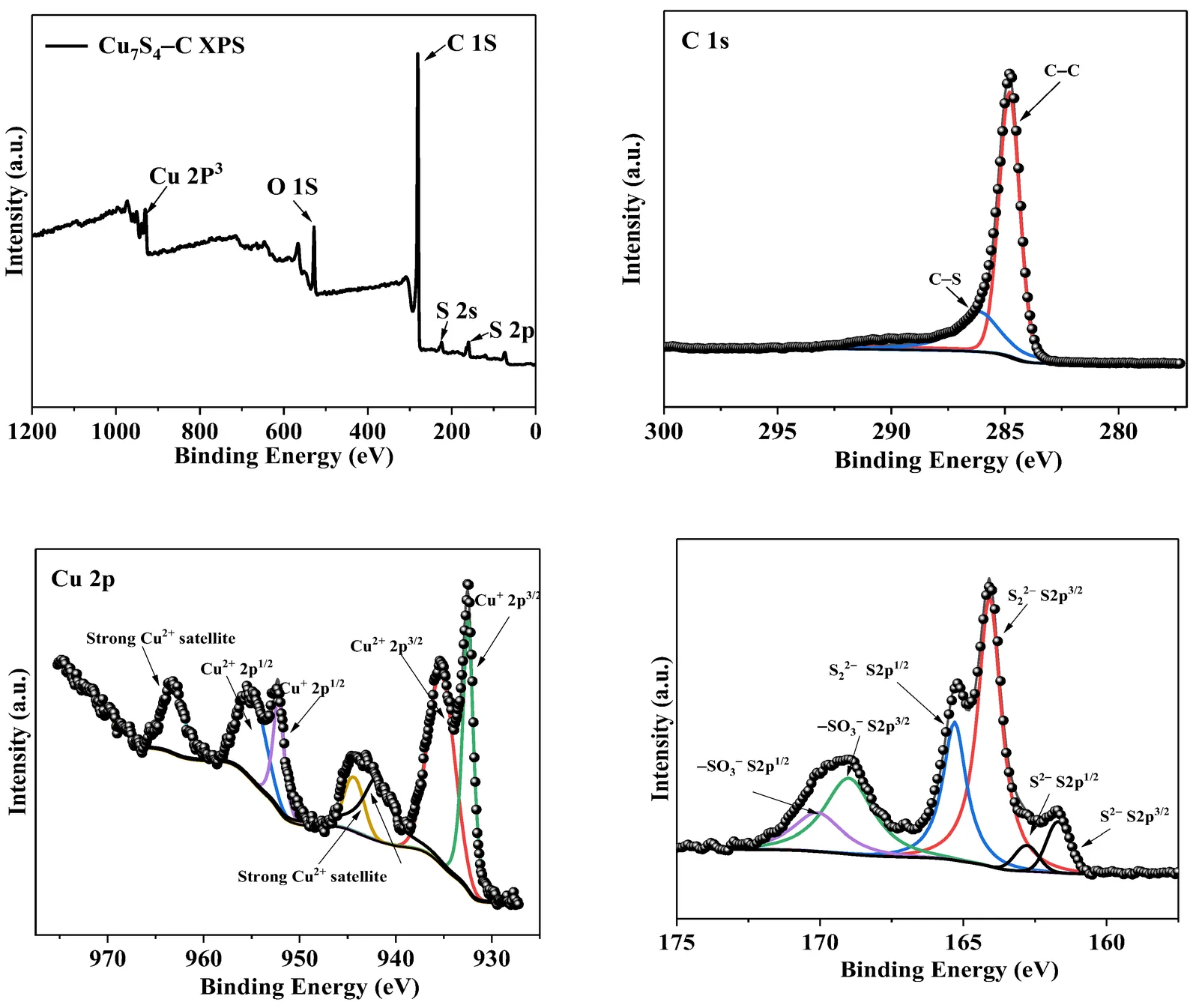
XPS spectra of Cu_7_S_4_-C material.

The surface valence structure and elemental composition of the prepared materials were investigated using X-ray photoelectron spectroscopy (XPS), and the results are presented in Figure 2, Figure 3 and Figure 4. The XPS results depicted in Figure 2 reveal the presence of elements (C, Co, O, and S) within the Co_3_S_4_-C material, with no detection of additional contaminants. Within the range of the high-resolution C1s spectrum illustrated in Figure 2, the two fitted peaks situated at 284.8 eV and 286.28 eV are assigned to C-C and C-S bonds, respectively. Furthermore, the high-resolution S 2p spectrum exhibited six distinct peaks, including two peaks at 162.31 and 163.41 eV attributed to S^2−^, two peaks at 164.07 and 165.32 eV corresponding to S_2_^2−^, and two peaks at 168.92 and 170.02 eV associated with the sulfonic acid group (–SO_3_^−^) [17]. In the high-resolution XPS spectrum of Co 2p, the peaks located at binding energies of 778.60 eV and 793.58 eV are assigned to the main peaks of Co^3+^ state (Co2p_3/2_ and Co2p_1/2_), while the peaks located at binding energies of 781.92 eV and 797.71 eV are attributed to Co^2+^ state (Co2p_3/2_ and Co2p_1/2_), and the satellite peaks of Co^2+^ and Co^3+^ are at 786.32 eV and 802.88 eV. The characteristic peaks of XPS binding energy mentioned above confirm the simultaneous existence of Co^2+^ and Co^3+^ in Co_3_S_4_-C material [18,19].

The XPS results in Figure 3 show the presence of elements (C, Ni, O and S) in the Ni_3_S_2_-C material, with no other contaminants detected. In the high-resolution C1s spectrum, the two fitted peaks at 284.8 eV and 285.71 eV correspond to the C-C and C-S states, respectively. The high-resolution XPS spectra of S 2p spectrum exhibited only four distinct peaks, two peaks at 162.25 eV and 163.44 eV attributed to S^2−^, two peaks at 164.08 eV and 165.26 eV corresponding to S_2_^2−^. Additionally, in the high-resolution XPS spectrum of the Ni element, the peaks located at 856.30 eV and 873.70 eV are assigned to the main peaks of Ni^2+^ 2p_3/2_ and Ni^2+^ 2p_1/2_, respectively. The Ni^2+^ satellite peaks, consisting of two single peaks at 859.88 eV and 876.99 eV, and the peaks located at 853.12 eV and 870.33 eV are assigned to the main peaks of Ni^0^ 2p_3/2_ and Ni^0^ 2p_1/2_, which confirm the presence of Ni^0^ and Ni^2+^ in the material [20,21].

The XPS results in Figure 4 show the presence of C, Cu and S elements in the Cu_7_S_4_-C material, with no other contaminants detected. In the high-resolution XPS of the C1s spectrum, the two fitted peaks at 286.14 eV and 284.8 eV correspond to the C-S and C-C states, respectively. The high resolution XPS of S 2p spectrum exhibits peaks at 161.69 eV and 162.79 eV, which are attributed to the S 2p_3/2_ and S 2p_1/2_ of copper metal sulfides. The peaks at 164.09 eV and 165.30 eV are assigned to the S 2p_3/2_ and S 2p_1/2_ of the thiol structure C-S-H bond. Additionally, the peaks at 168.99 eV and 170.09 eV are attributed to the S 2p_3/2_ and S 2p_1/2_ of sulfite (–SO_3_^−^). In the high-resolution XPS spectrum of Cu 2p in Figure 4, the peaks located at 935.24 eV and 954.92 eV are assigned to the main peaks of Cu 2p_3/2_ and Cu 2p_1/2_, respectively. The satellite peaks, consisting of a doublet at 944.32 eV and 941.97 eV and a singlet at 963.13 eV, confirm the presence of Cu^2+^ in the material [22]. Meanwhile, the peaks at 932.47 eV and 952.21 eV are likely the main peaks of Cu^+^.

**Figure 5:**
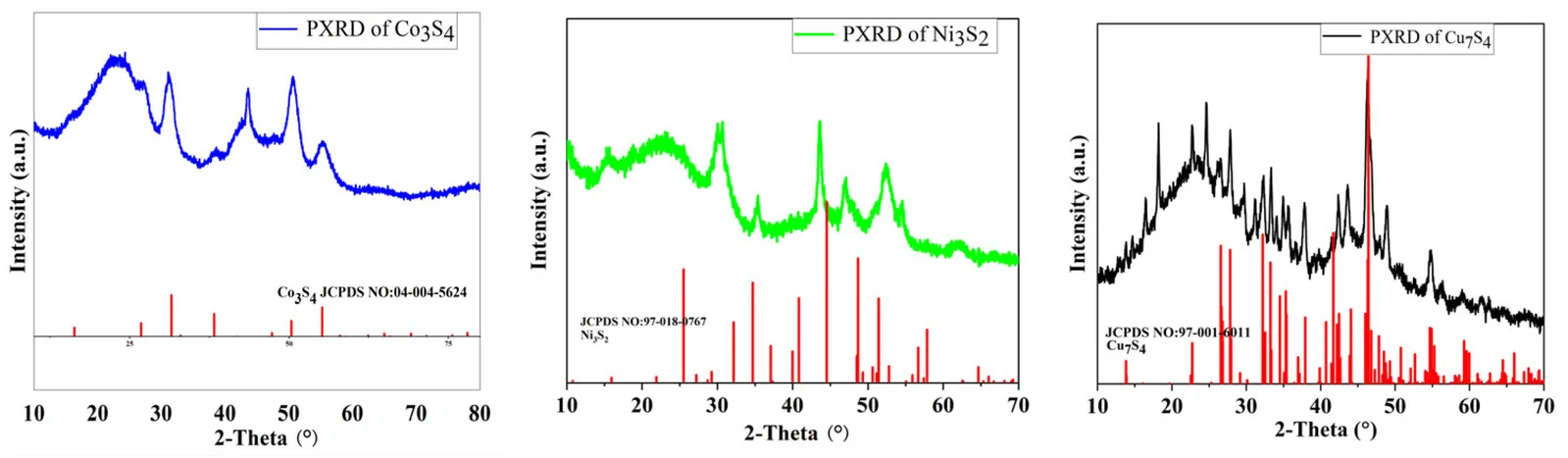
PXRD patterns of Co_3_S_4_-C, Ni_3_S_2_-C and Cu_7_S_4_-C.

The powder X-ray diffraction (PXRD) tests were investigated on the Co_3_S_4_-C, Ni_3_S_2_-C and Cu_7_S_4_-C materials for accurately determining the crystalline phase, and the patterns are depicted in Figure 5. For Co_3_S_4_-C, the diffraction peaks detected at 31.5°, 38.4°, 50.31°, and 55.30° were assigned to the (311), (400), (511), and (440) crystal planes of Co_3_S_4_ (PDF: 04-004-5624), respectively. In the PXRD spectrum of Ni_3_S_2_-C material, the diffraction peaks at 29.9°, 30.9°, 38.8°, 43.8°, 47.4°, 52.5°, 56.3° and 63.5° were assigned to the (110), (022), (130), (023), (132), (061), (133) and (114) crystal planes of Ni_3_S_2_ (PDF: 97-018-0767), respectively. The PXRD pattern of Cu_7_S_4_-C closely matches that of the orthorhombic Cu_7_S_4_ (PDF: 97-001-6011). Based on the analysis results of XPS and PXRD data, the composition of the prepared materials can be determined; they are composites formed by the combination of Co_3_S_4_, Ni_3_S_2_, and Cu_7_S_4_ with carbon, respectively.

**Figure 6:**
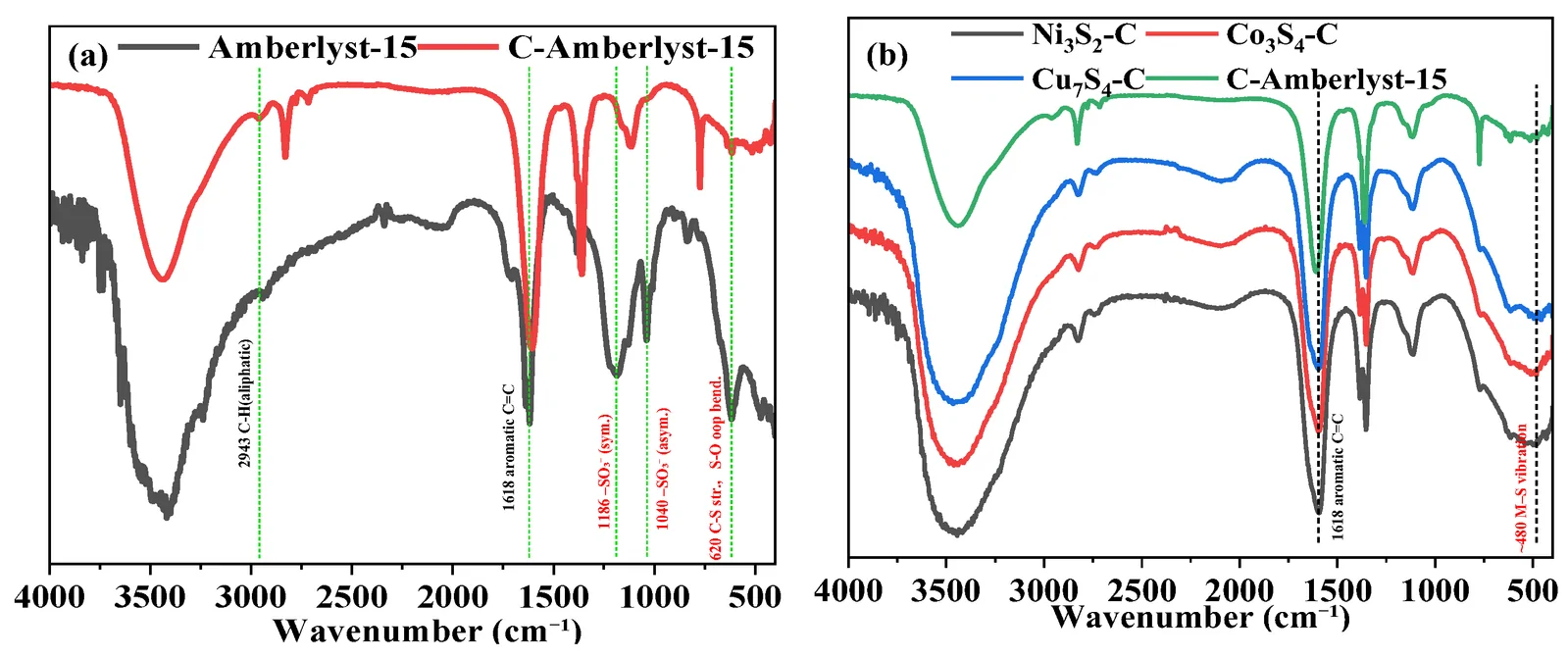
(**a**) FTIR spectra of Amberlyst 15, and C-Amberlyst 15, and (**b**) Co_3_S_4_-C, Ni_3_S_2_-C, Cu_7_S_4_-C, and C-Amberlyst15.

The surface functional groups and the in situ formation of metal–sulfur bonds were further examined by Fourier-transform infrared (FTIR) spectroscopy. As shown in Figure 6a, the pristine Amberlyst 15 resin exhibits two characteristic absorption bands at approximately 1186 and 1040 cm^−1^, assigned to the asymmetric and symmetric S=O stretching vibrations of the –SO_3_H groups, respectively. The band near 620 cm^−1^ is attributed to C–S stretching and S–O out-of-plane bending vibrations of the sulfur-containing carbon matrix. After pyrolysis at 800 °C, all three bands disappear almost completely, confirming quantitative decomposition of the sulfonic acid groups and the release of sulfur for in situ sulfidation. Figure 6b compares the FTIR spectra of the metal-free carbonized Amberlyst 15 (C-Amberlyst) with those of Co_3_S_4_-C, Ni_3_S_2_-C and Cu_7_S_4_-C. Compared with C-Amberlyst, all three composites exhibit a broad new absorption band in the 400–650 cm^−1^ region that is essentially absent in C-Amberlyst, which is assigned to the M–S stretching vibrations of the in situ-formed sulfide phases. The appearance of this low-frequency band in all three composites confirms the successful in-situ formation of Co_3_S_4_, Cu_7_S_4_ and Ni_3_S_2_ phases within the carbon matrix. The simultaneous appearance of these M–S bands with the preservation of the 1618 cm^−1^ aromatic C=C band demonstrates that the sulfur released from the –SO_3_H groups of Amberlyst 15 has reacted in situ with the anchored metal ions, rather than being washed away during impregnation.

The in situ formation of metal sulfides can be rationalized by the combined TGA–DTG, FTIR, XPS and PXRD evidence. During pyrolysis, the –SO_3_H groups of Amberlyst 15 decompose in the 200–330 °C range (Figure S1), releasing sulfur-containing species (SO_2_, H_2_S or elemental sulfur) that react in situ with the anchored transition-metal ions (Co^2+^, Ni^2+^, Cu^2+^) to form the corresponding metal sulfides (Co_3_S_4_, Ni_3_S_2_, Cu_7_S_4_) at 800 °C. The disappearance of the 1186/1040 cm^−1^ –SO_3_H bands in the FTIR spectra, along with the band near 620 cm^−1^ assigned to C–S and S–O stretching (Figure 6a), and the concurrent appearance of the 400–650 cm^−1^ M–S band (Figure 6b), directly confirm this sulfur-release and sulfidation process. Meanwhile, the polystyrene-divinylbenzene backbone undergoes progressive carbonization above 330 °C, retaining the aromatic C=C framework (1618 cm^−1^) while forming a conductive sp^2^ carbon matrix that supports and disperses the in situ formed sulfide nanoparticles. This one-pot, template-free in situ sulfidation strategy ensures intimate M–S–C contact and uniform dispersion of the sulfide phase within the carbon support.

**Figure 7:**
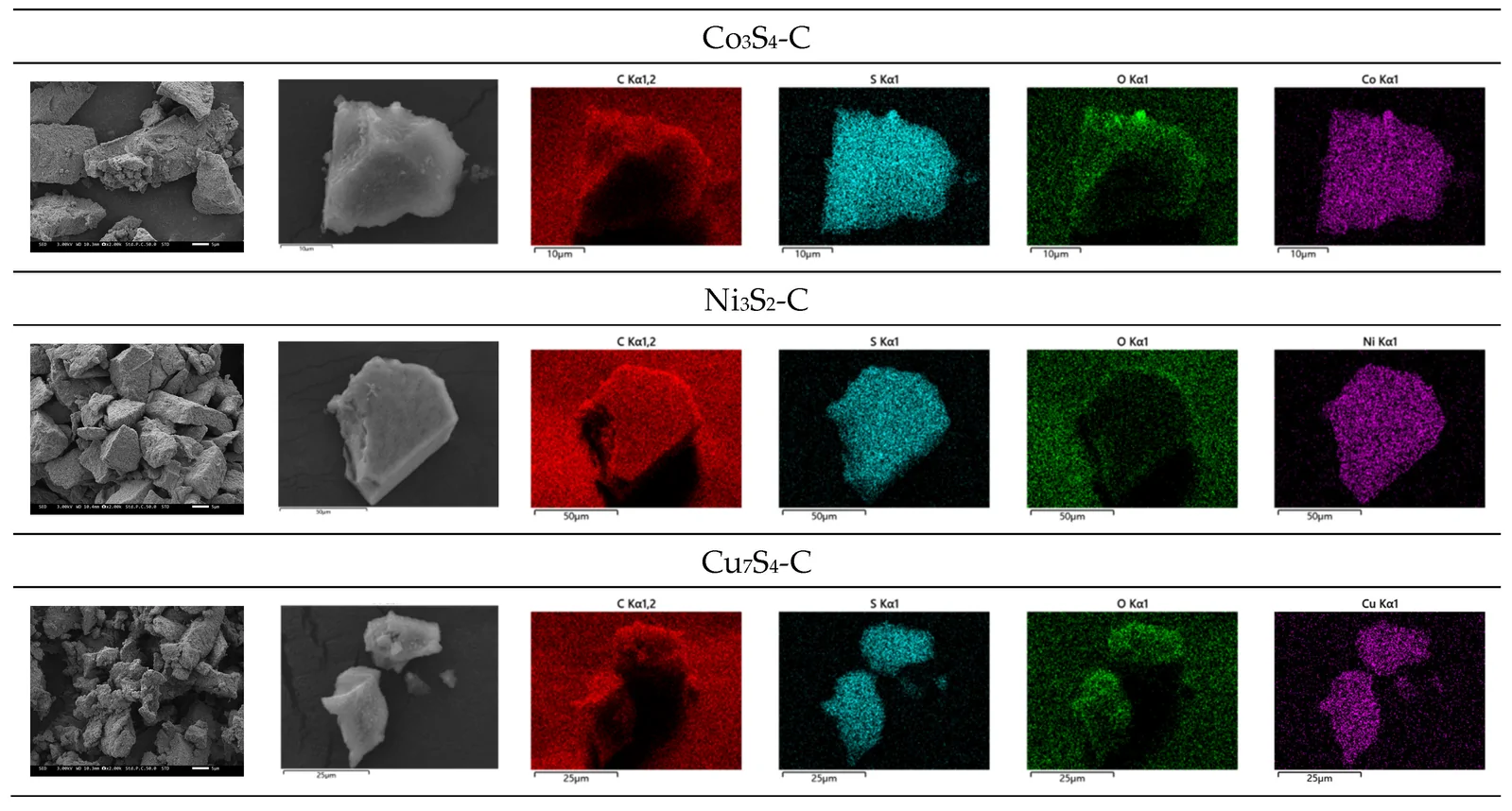
SEM and mapping images of Co_3_S_4_-C, Ni_3_S_2_-C and Cu_7_S_4_-C materials.

Scanning electron microscopy (SEM) images (Figure 7) of the Co_3_S_4_-C, Ni_3_S_2_-C, and Cu_7_S_4_-C materials reveal that all of the prepared samples are composed of nanoscale particles that form a bulk structure. Mapping images (Figure 7) exhibit that all elements in the Co_3_S_4_-C, Ni_3_S_2_-C, and Cu_7_S_4_-C materials are evenly dispersed. Quantitative EDS mapping analysis (Tables S2–S4 in the Supplementary Materials) further confirms the elemental composition and uniform dispersion of C, S and metal elements in the composites; the content of metal cobalt, nickel, and copper is 3.21 wt%, 2.48 wt% and 4.02 wt%, respectively.

**Figure 8:**
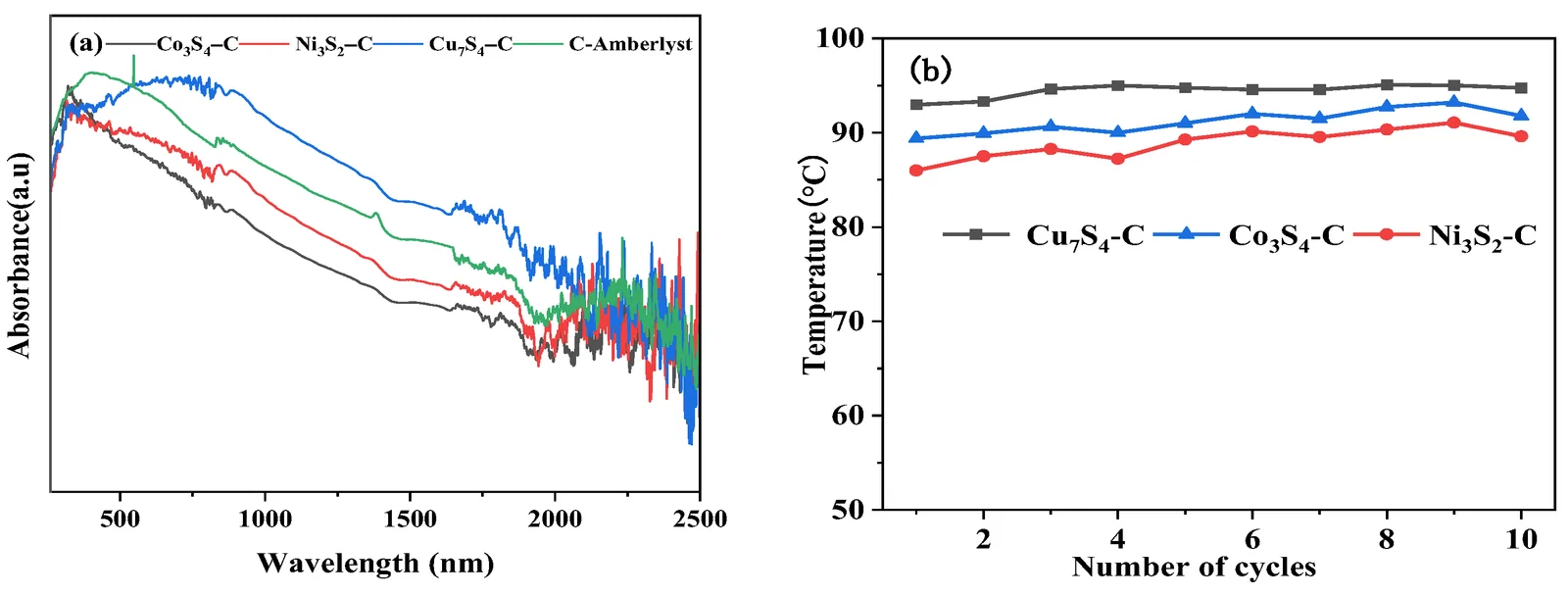
(**a**) UV-Vis-NIR absorption spectra of Co_3_S_4_-C, Ni_3_S_2_-C, Cu_7_S_4_-C materials and C-Amberlyst 15, (**b**) the maximum surface temperature of dry Co_3_S_4_-C, Ni_3_S_2_-C and Cu_7_S_4_-C materials over 10 times under a standard solar irradiation.

The ability to absorb light is crucial for solar thermal conversion materials. The measurement of the UV-Vis-NIR absorption spectra is necessary for this purpose. As shown in Figure 8a, Co_3_S_4_-C, Ni_3_S_2_-C, and Cu_7_S_4_-C all exhibit strong UV–Vis–NIR absorption across the 250–2500 nm range, indicating their potential for enhanced light utilization efficiency. The slurry, prepared by uniformly mixing 20 mg of transition metal sulfide/carbon composite material with polyvinyl alcohol, was applied evenly onto the surface of a 3 cm × 3 cm square wooden cellulose sponge. Then, the surface was irradiated with a simulated light source at a light intensity of 1 kW·m^−2^, and the maximum surface temperature under dry conditions was measured with an infrared imaging camera. After the surface temperature reached its maximum, irradiation was continued for three hours. This operation was repeated 10 times; the maximum surface temperature was recorded each time, and the recorded temperatures are presented in Figure 8b. The average value of maximum surface temperatures for Co_3_S_4_-C, Ni_3_S_2_-C, and Cu_7_S_4_-C materials was found to be 91 °C, 89 °C, and 94 °C, in that order. From this, it can be inferred that the photo-thermal conversion efficiency of Cu_7_S_4_-C material was the highest among the three prepared materials, indicating that higher interfacial water evaporation efficiency can be achieved. Moreover, over the 10 cycles, the maximum temperature of all materials did not decrease, indicating excellent light tolerance, no photothermal degradation, and good optical stability. These properties enable the materials to maintain high efficiency and operate for extended periods under illumination.

### 3.2. Solar-Driven Interfacial Water Evaporation Performance

**Figure 9:**
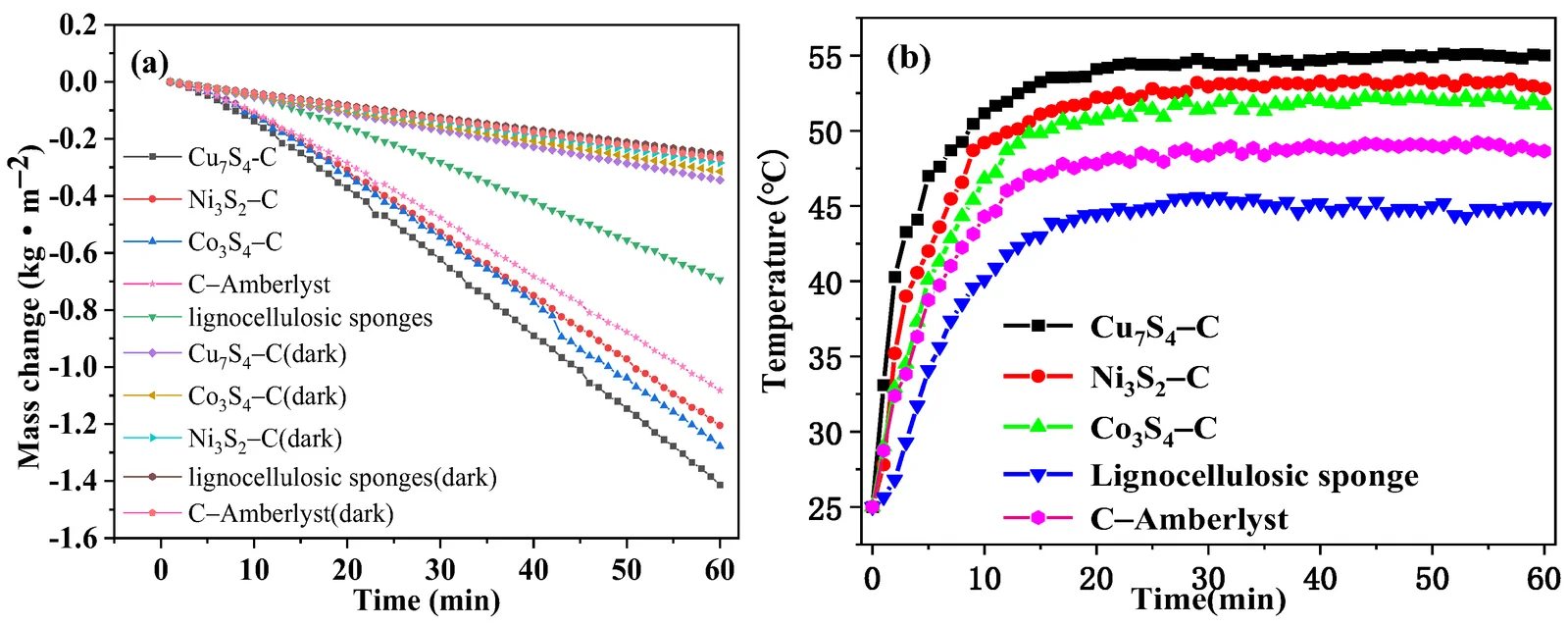
(**a**) The water mass change over different evaporators with time, and (**b**) change of surface temperature of different evaporators with time under a standard solar irradiation.

Based on the characterization of the three previously prepared transition metal sulfide/carbon composites, they exhibit a significant absorption effect across the full spectrum and remarkable photothermal conversion performance. Consequently, the evaporation performance of photothermal evaporation devices made of these composite materials in indoor environments was explored in more depth. Figure 9a presents the mass change of water within 60 min for Cu_7_S_4_-C, Ni_3_S_2_-C, and Co_3_S_4_-C photothermal materials, as well as a lignocellulosic sponge, under 1 Sun illumination and dark conditions. Under 1 Sun illumination, the one-hour water evaporation capacities of evaporators composed of Cu_7_S_4_-C, Ni_3_S_2_-C, Co_3_S_4_-C, and the lignocellulosic sponge were 1.58, 1.35, 1.47, 1.21, and 0.82 kg·m^−2^, respectively. These results indicate that introducing photothermal conversion materials significantly enhances the evaporation rate at the evaporator surface, with the Cu_7_S_4_-C system showing the most pronounced improvement. This enhancement can be rationalized by a synergistic photothermal mechanism involving two coupled channels. First, the Amberlyst-derived sp^2^ carbon matrix provides broad-spectrum absorption across the UV–vis–NIR range and relaxes photoexcited π-electrons into phonons; Second, the in-situ formed metal sulfides (Co_3_S_4_, Cu_7_S_4_, Ni_3_S_2_) introduce an additional NIR absorption band assigned to their localized surface plasmon resonance (LSPR). The intimate M–S–C interface established during in situ sulfidation at 800 °C enables rapid injection of plasmon-generated hot carriers into the conductive carbon network. The comparison with the metal-free C-Amberlyst control further demonstrates that the sulfide phase contributes additional NIR absorption and photothermal performance beyond the intrinsic response of the carbon support [23,24]. Under dark conditions, the corresponding one-hour water evaporation capacities were 0.35, 0.29, 0.32, 0.27, and 0.26 kg·m^−2^ for Cu_7_S_4_-C, Ni_3_S_2_-C, Co_3_S_4_-C, C-Amberlyst-15, and the lignocellulosic sponge, respectively. Under 1 sun irradiance (1 kW·m^−2^), an infrared thermal imager was employed to record real-time temperature variations on the evaporator surface, while simultaneous mass changes during evaporation were monitored. Figure 9b displays the surface temperature profiles of photothermal materials Co_3_S_4_-C, Ni_3_S_2_-C, Cu_7_S_4_-C and lignocellulosic sponge under 1 sun irradiation over 60 min. All materials exhibited rapid temperature increases upon illumination, stabilizing after 30 min. The highest surface temperatures reached 53.2 °C for Cu_7_S_4_-C, 52.3 °C for Ni_3_S_2_-C, 52.8 °C for Co_3_S_4_-C, C-Amberlyst-15 for 48.9 °C, and 45.1 °C for lignocellulosic sponge, respectively. These results indicate that the introduction of photothermal conversion materials significantly increases the temperature on the surface of the evaporator, with the most pronounced effect achieved after the addition of Cu_7_S_4_-C material.

The solar-to-vapor conversion efficiency (η) was calculated according to the following equations [25,26]:

*η* = *ν*_net_(*Q* + Δ*H*_LV_)/*E*_in_; Δ*H*_LV_ = 1.91846 × 10^3^ [*T*_1_/(*T*_1_ − 33.9)]^2^; *Q* = *c*(*T*_1_ − *T*_0_)

In this equation, *η* represents the evaporation efficiency, *ν*_net_ is the net water evaporation rate (kg·m^−2^·h^−1^), Δ*H*_LV_ is the total enthalpy required for the transition of water from its liquid to vapor phase (J·g^−1^), *T*_0_ is the original temperature of water (K), set at 298.15 K; and *T*_1_ is the stable surface temperature of the sample evaluated using an infrared camera (K), and *Q* is the sensible heat of water of unit mass, and *c* is the specific heat of water (4.2 J·g^−1^·K^−1^), *E*_in_ is the incident solar energy, 1.0 kW·m^−2^. The final calculation results demonstrated that the evaporation efficiency of the evaporators constructed from Cu_7_S_4_-C, Ni_3_S_2_-C, Co_3_S_4_-C, and C-Amberlyst-15 was 85.7%, 73.8%, 80.1%, and 65.2%, respectively, while the evaporation efficiency of the evaporator made from lignocellulosic sponge was only 38.7%. Among all the evaporators examined in this study, the one composed of Cu_7_S_4_-C material exhibited the highest evaporation rate and the best evaporation efficiency, further confirming that Cu_7_S_4_-C material is an excellent photothermal conversion material.

**Figure 10:**
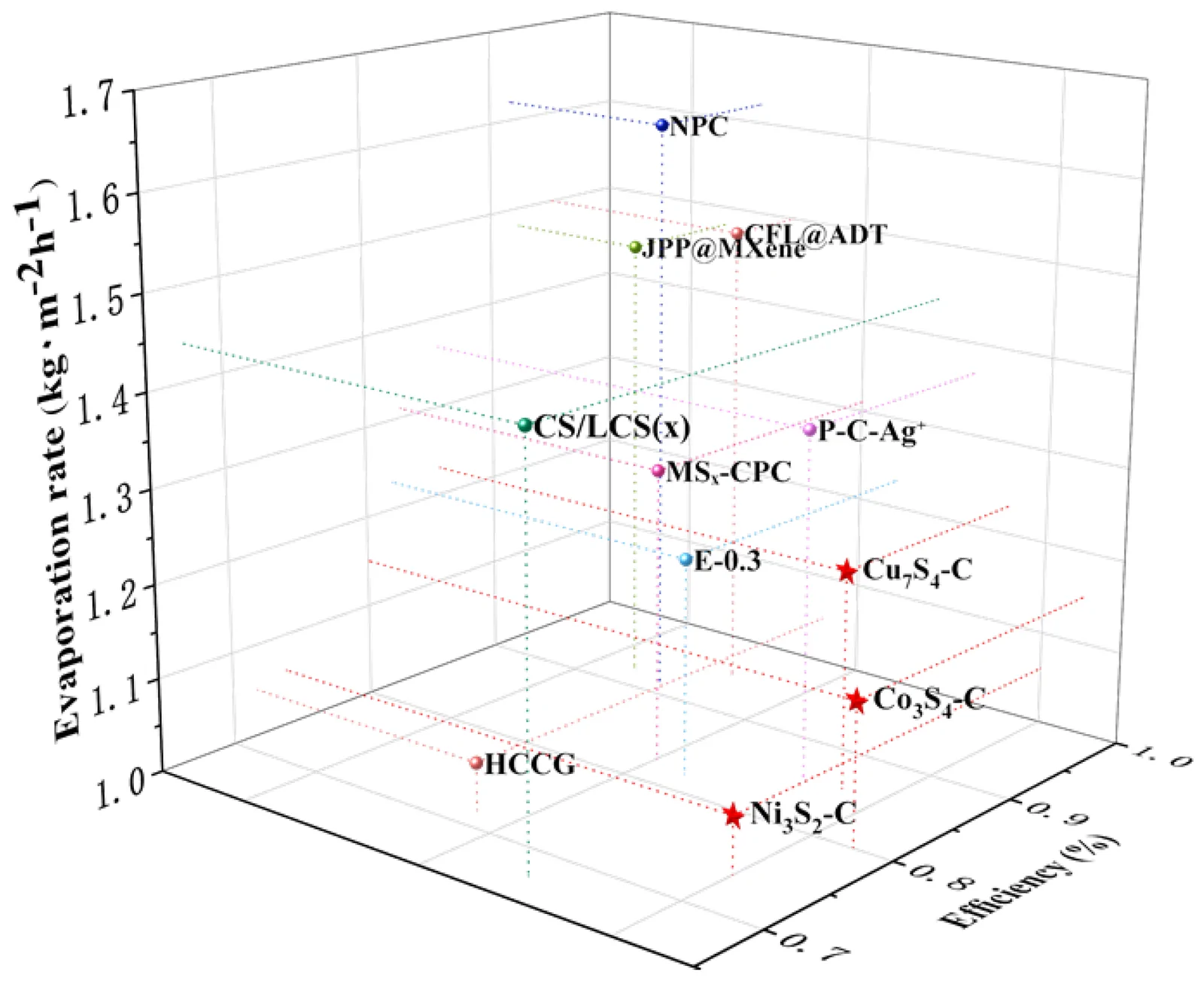
Solar evaporation performance based on transition-metal sulfide/carbon composites in this work compared with other waste-derived materials under 1 sun.

To comprehensively evaluate the solar evaporation performance of the as-fabricated transition-metal sulfide/carbon composites, we benchmarked the evaporation rate and photothermal efficiency of Cu_7_S_4_-C, Ni_3_S_2_-C and Co_3_S_4_-C against a series of previously reported evaporators under 1 sun illumination (1 kW·m^−2^), as summarized in Figure 10 and Table S5. The reference materials including JPP@MXene (1.48 kg·m^−2^·h^−1^, 92.3%) [27], NPC (1.62 kg·m^−2^·h^−1^, 91.6%) [28], CFL@ADT (1.5 kg·m^−2^·h^−1^, 95%) [29], HCCG (1.05 kg·m^−2^·h^−1^, 71.7%) [30], MS_x_-CPC (1.31 kg·m^−2^·h^−1^, 82.7%) [31], E-0.3 (1.23 kg·m^−2^·h^−1^, 82%) [32], CS/LCS(x) (1.44 kg·m^−2^·h^−1^, 67.5%) [33], P-C-Ag^+^ (1.367 kg·m^−2^·h^−1^, 85.66%) [34] exhibit evaporation rates ranging from 1.05 to 1.62 kg·m^−2^·h^−1^ with photothermal efficiencies spanning 67.5–95%. Among these reported systems, CFL@ADT delivers a high efficiency of 95% yet a moderate evaporation rate of 1.5 kg·m^−2^·h^−1^, while NPC achieves a competitive evaporation rate of 1.62 kg·m^−2^·h^−1^ but with a slightly lower efficiency of 91.6%. The Cu_7_S_4_-C composite developed in this work achieves a net photothermal efficiency of 85.7% together with an evaporation rate of 1.23 kg·m^−2^·h^−1^, a combination that compares favorably with most referenced evaporators in terms of overall performance. The other two sulfide/carbon composites, Ni_3_S_2_-C (1.06 kg·m^−2^·h^−1^, 73.8%) and Co_3_S_4_-C (1.15 kg·m^−2^·h^−1^, 80.1%), also display comparable or superior solar evaporation capacities relative to most reported counterparts. These comparative results unambiguously verify that the Cu_7_S_4_-C architecture integrates excellent water evaporation capacity and ultrahigh light-to-heat conversion efficiency, demonstrating great promise as a high-performance solar evaporator for practical solar-driven interfacial evaporation applications.

**Figure 11:**
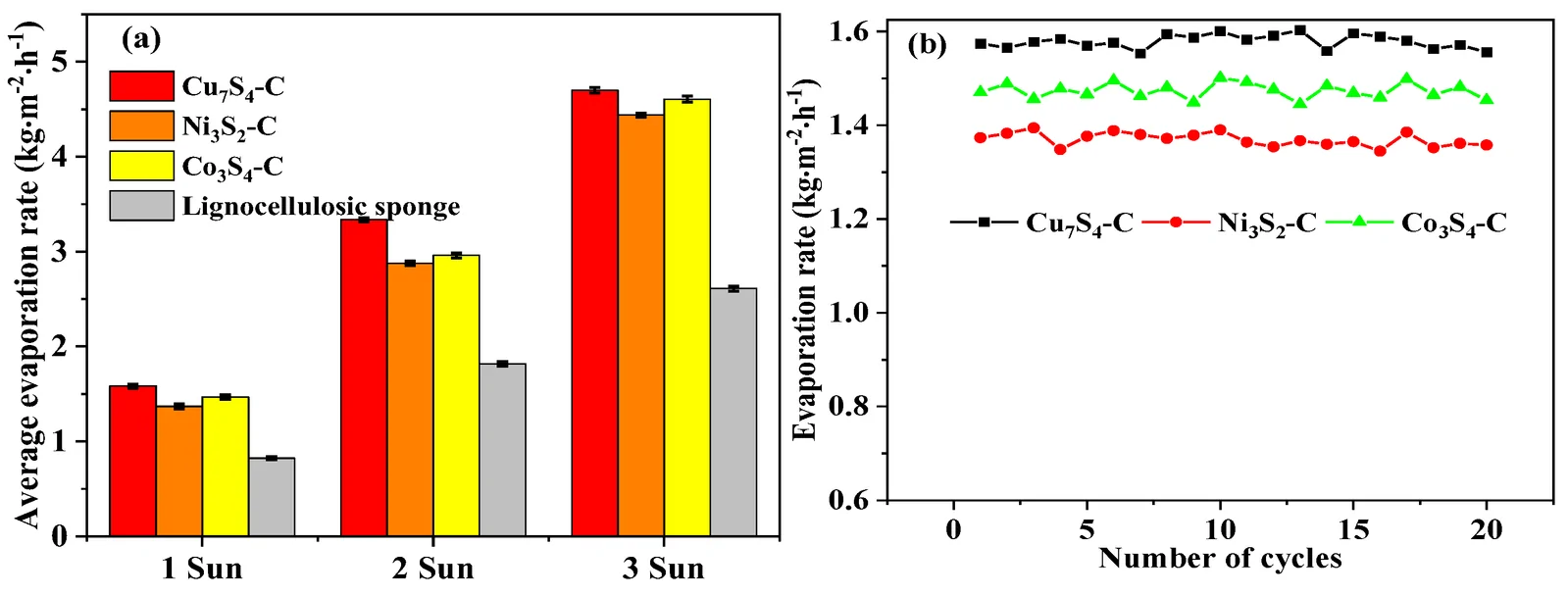
(**a**) The average apparent water evaporation rate of photothermal materials under 1, 2, and 3 Sun irradiation, (**b**) the evaporation rates of Cu_7_S_4_-C, Ni_3_S_2_-C, and Co_3_S_4_-C for 20 cycles.

The average apparent evaporation rates of composite materials of transition metal sulfides and carbon under various light intensities were also measured. As depicted in Figure 11a, 1 Sun: Cu_7_S_4_-C (1.58 kg·m^−2^·h^−1^), Ni_3_S_2_-C (1.35 kg·m^−2^·h^−1^), Co_3_S_4_-C (1.47 kg·m^−2^·h^−1^), and lignocellulosic sponge (0.82 kg·m^−2^·h^−1^); 2 Suns: Cu_7_S_4_-C (3.34 kg·m^−2^·h^−1^), Ni_3_S_2_-C (2.88 kg·m^−2^·h^−1^), Co_3_S_4_-C (2.96 kg·m^−2^·h^−1^), and lignocellulosic sponge (1.81 kg·m^−2^·h^−1^); 3 Suns: Cu_7_S_4_-C (4.70 kg·m^−2^·h^−1^), Ni_3_S_2_-C (4.44 kg·m^−2^·h^−1^), Co_3_S_4_-C (4.61 kg·m^−2^·h^−1^), and lignocellulosic sponge (2.61 kg·m^−2^·h^−1^). Based on the quantitative analysis of evaporation rates under 1, 2, and 3 suns, the linear relationship between evaporation rate and light intensity is generally excellent for the transition metal sulfide/carbon composites, but markedly poorer for the neat lignocellulosic sponge.

As presented in Figure 11b, no significant decline in the apparent photothermal evaporation performance was observed for Cu_7_S_4_-C, Ni_3_S_2_-C, and Co_3_S_4_-C after 20 cycles, confirming their favorable stability and cycling performance.

**Figure 12:**
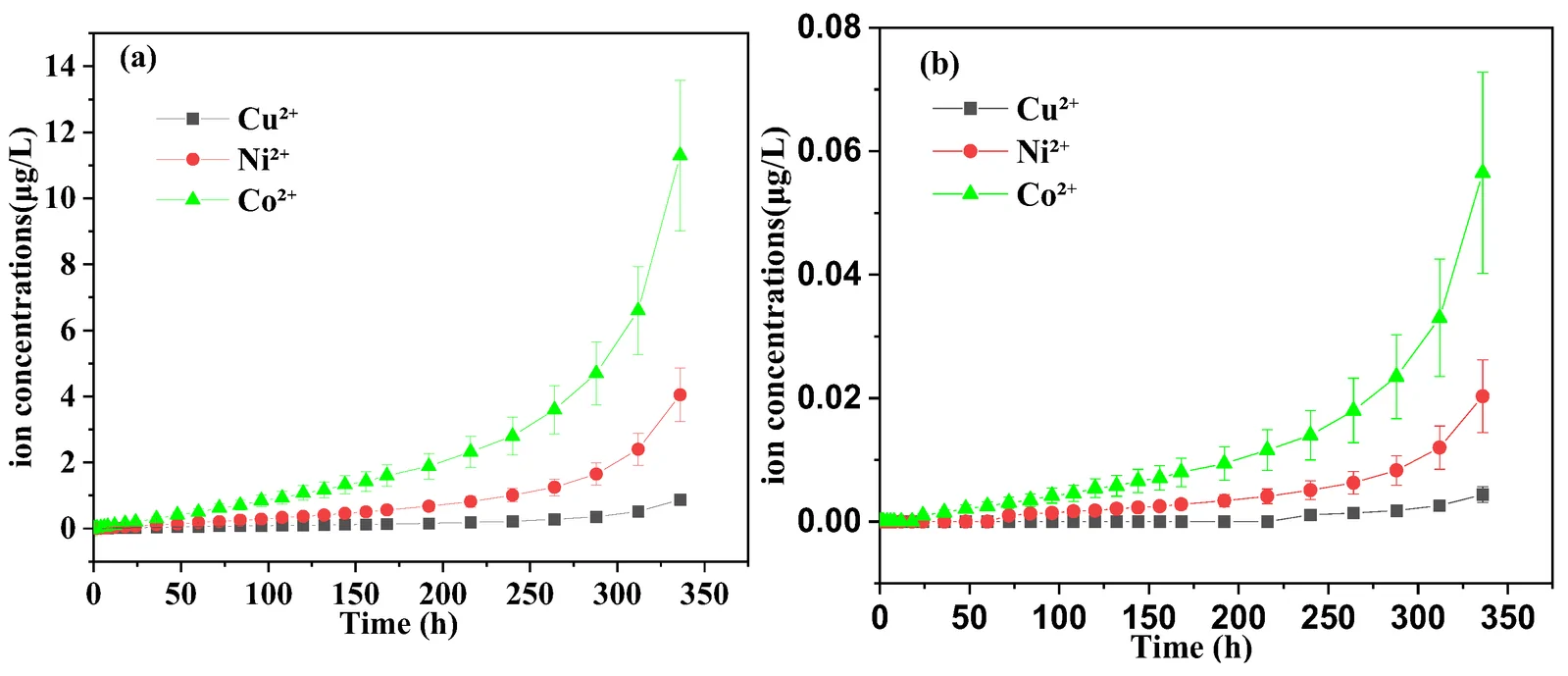
(**a**) Metal concentrations in environmental water bodies vary over time, and (**b**) Metal concentrations in condensed water vary over time.

Figure 12 shows the variation curves of Cu^2+^, Ni^2+^, and Co^2+^ concentrations in the environmental water body (a) and condensed water (b) during continuous operation for 336 h with Cu_7_S_4_-C, Ni_3_S_2_-C, and Co_3_S_4_-C photothermal materials under a 3 × 3 cm^2^ evaporator, 1000 mL environmental water, and one-sun illumination. As shown in Figure 12a, the leaching concentrations of the three metals in the environmental water body continuously increased with time, rapidly increasing within 0–24 h and then gradually slowing down; however, no complete plateau appeared within 336 h, which was mainly attributed to the continuous release from the materials and the concentration effect caused by water evaporation. At 336 h, the Cu^2+^ concentration for Cu_7_S_4_-C reached 0.875 ± 0.176 μg/L, the Ni^2+^concentration for Ni_3_S_2_-C was 4.050 ± 0.810 μg/L, and the Co^2+^ concentration for Co_3_S_4_-C was 11.30 ± 2.28 μg/L. The metal leaching amounts of the three materials followed the order Cu < Ni < Co, consistent with their intrinsic stability order (Cu_7_S_4_ > Ni_3_S_2_ > Co_3_S_4_). The curves exhibited slight fluctuations at some time points (e.g., Cu was almost constant at 96–108 h), reflecting the unavoidable experimental uncertainty in ICP-MS measurements (RSD of approximately 10–20%).

As shown in Figure 12b, the metal ions in the condensed water mainly originated from physical entrainment during evaporation; their concentrations were directly related to those in the environmental water body but were much lower than the latter (entrainment rate of approximately 0.5%). In the early stage of the experiment (0–216 h), the Cu^2+^ and Ni^2+^ concentrations in the condensed water were both below the ICP-MS detection limits (Cu^2+^ < 0.001 μg/L, Ni^2+^ < 0.010 μg/L), and Co^2+^ was only slightly above the detection limit after 24 h. As the metal concentrations in the environmental water body increased, the metal concentrations in the condensed water gradually became detectable in the later stage. At 336 h, the Cu^2+^, Ni^2+^, and Co^2+^ concentrations were 0.0044 ± 0.0013 μg/L, 0.0203 ± 0.0059 μg/L, and 0.0565 ± 0.0163 μg/L, respectively. The metal concentrations in the condensed water at all time points were far below the limits of the Standards for Drinking Water Quality (GB 5749-2022, China [35]) (Cu^2+^ 1.0 mg/L, Ni^2+^ 0.02 mg/L); the measured values for Cu^2+^ and Ni^2+^ were only 0.00044% and 0.10% of the limits, respectively. Although the Standards for Drinking Water Quality (GB 5749-2022) do not set a limit for Co, its concentration (0.0565 μg/L) was only 0.113% and 0.00565% of the cobalt limits for centralized drinking-water sources specified in GB/T 14848-2017 (Class III groundwater, 50 μg/L) [36] and GB 3838-2002 (surface water, 1.0 mg/L) [37], respectively, confirming the safety of the produced water [38].

To further verify the safety of the condensed water and to assess the environmental risk of the leached metals, the 336-h concentrations were evaluated against drinking water standards and aquatic protection benchmarks. As summarized in Table S6, the Cu^2+^, Ni^2+^, and Co^2+^ concentrations in the condensed water were 0.0044, 0.0203, and 0.0565 μg/L, respectively, all far below the corresponding limits and therefore classified as safe. For the environmental water body, the risk quotient (RQ = MEC/PNEC) values in Table S7 were 0.875 for Cu^2+^, 0.506 for Ni^2+^, and 2.83 for Co^2+^ [38]. According to the risk classification (RQ < 0.1, negligible; 0.1 ≤ RQ < 1, low; RQ ≥ 1, potential), both Cu and Ni were at a low-risk level, while Co^2+^ showed a potential risk (RQ > 1) under the conservative closed-water scenario [39]. However, the Co^2+^ RQ is an extremely conservative estimate based on a 1000 mL closed water body with continuous evaporative concentration over 336 h. In actual open water bodies, dilution and replenishment would significantly reduce the concentration; for example, if the water volume is 5 L, the Co^2+^ concentration would decrease to approximately 1.13 μg/L, and the RQ would decrease to 0.28, indicating negligible risk.

Combining Figure 12a,b with Tables S6 and S7, it can be concluded that under the conservative conditions of a 3 × 3 cm^2^ evaporator, 1000 mL of limited water, and 336 h of continuous operation, the Cu^2+^ and Ni^2+^ concentrations in the environmental water body were at a low-risk level. Although the Co concentration was relatively the highest, the condensed water quality remained safe, and the impact of metal leaching on the produced water quality was negligible. Among the three materials, Cu_7_S_4_-C exhibited the best environmental safety, followed by Ni_3_S_2_-C; Co_3_S_4_-C requires slight attention in long-term closed water bodies, but in actual open water bodies, dilution and replenishment effects will significantly reduce its environmental risk.

## 4. Conclusions

In summary, the utilization of solid waste Amberlyst 15 sulfonic acid resin, functioning as both a carbon source and a sulfur source, in conjunction with calcination of transition metal nitrates (copper, nickel, cobalt) at 800 °C, results in the production of Cu_7_S_4_, Ni_3_S_2_, and Co_3_S_4_ composite materials, which exhibit remarkable photothermal effects. The combination of these materials results in a synergistic effect, whereby the electron-phonon coupling effect of carbon materials is integrated with the LSPR exhibited by transition metal sulfides. This integration serves to enhance solar spectrum absorption and photothermal conversion efficiency. Evaporation experiments of distilled water showed that, under one-sun irradiation, the water net evaporation rates of evaporators constructed from Cu_7_S_4_-C, Ni_3_S_2_-C, and Co_3_S_4_-C reached 1.23, 1.06, and 1.15 kg·m^−2^·h^−1^, respectively, with corresponding net photothermal conversion efficiencies of 85.7%, 73.8%, and 80.1%. Furthermore, the results of 20-cycle evaporation tests reveal that the evaporation rates of Cu_7_S_4_-C, Ni_3_S_2_-C, and Co_3_S_4_-C exhibit no significant degradation after repeated use, demonstrating excellent long-term stability and reusability. In addition, the condensed water quality remained safe, with metal concentrations far below drinking water standards; Cu_7_S_4_-C and Ni_3_S_2_-C exhibited low environmental risk, while Co_3_S_4_-C showed a potential risk only under the conservative closed-water scenario. In open water bodies, dilution would reduce the risk to a negligible level. This work provides a new approach for the resource utilization of waste Amberlyst 15 ion exchange resin, which can even be extended to the recycling of other sulfur-containing resins, in line with the principle of sustainable development. Additionally, it offers a novel type of photothermal conversion material for the application of solar-driven interfacial water evaporation systems.

## Data Availability

The original contributions presented in this study are included in the article/Supplementary Materials. Further inquiries can be directed to the corresponding authors.

## Supplementary Materials

The following supporting information can be downloaded at: https://www.mdpi.com/article/10.3390/ma19194238/s1, Figure S1 (TGA-DTG curves of Amberlyst 15 resin); Table S1 (elemental analysis of Amberlyst 15); Tables S2–S4 (quantitative EDS results of Ni_3_S_2_-C, Cu_7_S_4_-C, and Co_3_S_4_-C); Table S5 (comparison of solar evaporation performance with reported evaporators under 1 Sun); Table S6 (comparison of metal concentrations in condensed water with drinking water standards); Table S7 (environmental risk assessment based on RQ values); Table S8 (evaporation performance data under 1 Sun); Table S9 (synthesis parameters of the three composites).
