# Supplementary material for: Transition-Metal Sulfide/Carbon Composites Derived from Recycled Sulfonated Resin as High-Photothermal-Conversion Materials for Solar-Driven Interfacial Evaporation
Source: Materials (Basel). 2026 Oct 6;19(19):4238. doi: 10.3390/ma19194238 (PMC13665918; doi:10.3390/ma19194238)
Supplement: Supplementary file 1 [file materials-19-04238-s001.zip › materials-4543886-supplementary.pdf]

Supplementary Materials

Transition-Metal Sulfide/ Carbon Composites Derived from Recycled Sulfonated Resin as High Photothermal-Conversion Materials for Solar-Driven Interfacial Evaporation

Gexin Zhao[a, b], Meiyan Guo[b], Ao Liu[b], Jianming Tang[b], Gang Xiong[b*], Guangwen Xu[a, b*]

[a]School of Chemistry and Chemical Engineering, Liaoning University of Science and Technology, Anshan, 114051, China.

[b]Key Laboratory on Resources Chemicals and Materials of Ministry of Education, Shenyang University of Chemical Technology, Shenyang 110142, China.

*Corresponding authors: Contact info: xg@syuct.edu.cn, (G. Xiong); gwxu@syuct.edu.cn (G. W. Xu)

Table S1. Elemental analysis of Amberlyst 15 resin

Sample	C(%)	H(%)	S(%)	O(%)
Amberlyst 15	43.88	5.35	18.26	32.51

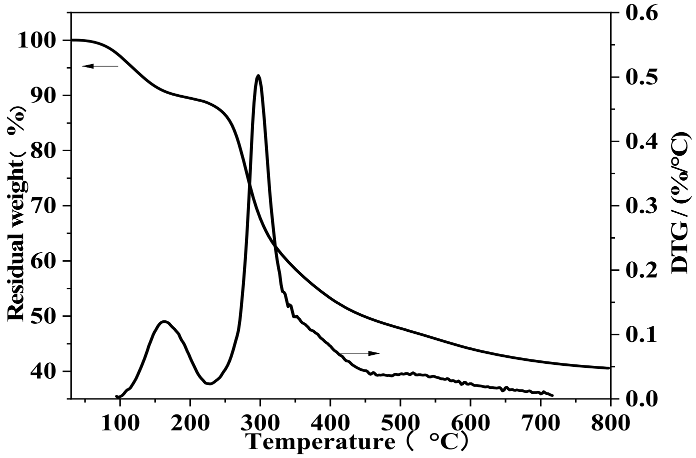

Figure S1. TGA and DTG curves of Amberlyst 15 resin.

The thermal decomposition behavior of the Amberlyst 15 resin was investigated

using thermogravimetric analysis (TGA) and derivative thermogravimetry (DTG), and the results are presented in Figure S1. The resin exhibited a total weight loss of 59.47% from 30 to 797 °C, with a final residue of 40.56%. The decomposition proceeded in four stages. In the first stage (30–200 °C), a weight loss of 10.51% was observed, accompanied by a weak DTG peak at ca. 114.5 °C (0.120 % $°C^{-1}$), which is attributed to the removal of physically adsorbed water and residual solvent. The second stage (200–330 °C) was the main decomposition region, in which the mass decreased from 89.53% to 61.31%, corresponding to a weight loss of 28.22% and accounting for 47.5% of the total weight loss. The DTG curve reached its maximum at 279.6 °C with a value of 0.502 % $°C^{-1}$ (ca. 5.02 % min^{-1}), mainly associated with the decomposition of sulfonic acid groups and the initial cleavage of the polystyrene-divinylbenzene backbone. This process releases sulfur-containing species that can react in situ with transition-metal ions to form metal sulfides. The third (330–500 °C) and fourth (500-797 °C) stages showed weight losses of 13.50% and 7.24%, respectively, corresponding to further degradation of the polymer framework and progressive carbonization/condensation of the carbon residue. These results indicate that Amberlyst 15 undergoes significant decomposition above 200 °C and is almost fully carbonized at 800 °C, supporting the use of 800 °C as the pyrolysis temperature. In addition, the high residual mass of 40.56% at 797 °C suggests that the resin can serve as an effective carbon precursor and support for the resulting photothermal composites. Therefore, the TGA-DTG results confirm that the selected 800 °C pyrolysis treatment is sufficient to decompose the sulfonic acid groups, release sulfur for in situ sulfidation, and carbonize the polymer matrix into carbon supports for the Cu_7S_4-C, Ni_3S_2-C, and Co_3S_4-C photothermal materials.

Table S2. Ni_3S_2-C distribution total number

**Elements**	**Line type**	**k ratio**	**Wt%**	**Wt% Sigma**	**At%**
C	K series	1.32485	79.68	0.17	86.15
O	K series	0.05347	15.28	0.17	12.40
S	K series	0.04929	1.83	0.02	0.74
Ni	K series	0.07941	3.21	0.06	0.71
**Total:**			100.00		100.00

Table S3. Cu_7S_4-C distribution total number

Elements	Line type	k ratio	Wt%	Wt% Sigma	At%
C	K series	1.20779	76.97	0.20	84.18
O	K series	0.06163	17.50	0.19	14.37
S	K series	0.03885	1.51	0.02	0.62
Cu	K series	0.09132	4.02	0.08	0.83
**Total:**			100.00		100.00

Table S4. Co_3S_4-C distribution total number

Elements	Line type	k ratio	Wt%	Wt% Sigma	At%
C	K series	1.15681	80.48	0.18	86.39
O	K series	0.05502	15.41	0.18	12.42
S	K series	0.06510	1.63	0.02	0.65
Co	K series	0.09994	2.48	0.04	0.54
**Total:**			100.00		100.00

Table S5. Solar evaporation performance based ontransition-metal sulfide/ carbon composites in this work compared with other waste-derived materials under 1 sun.

Type	Evaporation rate ($kg \cdot m^{-2} \cdot h^{-1}$)	Efficiency (%)	Reference
JPP@MXene	1.48	92.3%	[1]
NPC	1.62	91.6%	[2]
CFL@ADT	1.5	95%	[3]
HCCG	1.05	71.7%	[4]
MS_x-CPC	1.31	82.7%	[5]
E-0.3	1.23	82%	[6]
CS/LCS(x)	1.44	67.5%	[7]
P-C-Ag^+	1.367	85.66%	[8]
Cu_7S_4-C	1.23	85.7%	
Ni_3S_2-C	1.06	73.8%	This work
Co_3S_4-C	1.15	80.1%	

Note: It should be explicitly noted that the comparison in Figure 10 is not a like-for-like comparison. The literature data were obtained under different experimental conditions, including light source and intensity, evaporator area, water volume and salinity, ambient temperature and humidity, thermal insulation, water supply, dark-evaporation correction, surface-temperature measurement, and efficiency calculation. In particular, some reported efficiencies are gross values without dark-evaporation correction, whereas the efficiencies in this work are net values after subtracting dark evaporation. Therefore, Figure 10 andTable S5are provided only as a qualitative benchmark, and no strict ranking or claim of superiority is intended. A fair comparison would require all evaporators to be tested under identical conditions.

Table S6. Comparison of metal concentrations in condensed water with drinking water standards and safety assessment.

Metal	Source material	336 h concentration (μg/L)	Standard limit	Standard source	Percentage of limit	Safety conclusion
Cu	Cu_7S_4-C	0.0044	1.0 mg/L (1000 μg/L)	GB 5749-2022 (China) [9]	0.00044%	Far below limit, safe
Ni	Ni_3S_2-C	0.0203	0.02 mg/L (20 μg/L)	GB 5749-2022 (China)	0.10%	Far below limit, safe
Co	Co_3S_4-C	0.0565	50 μg/L (Class III); 1.0 mg/L (surface)	GB/T 14848-2017 [10]; GB 3838-2002 [11]	0.113%; 0.00565%	Far below limits, safe

Note: The Standards for Drinking Water Quality (GB 5749-2022) do not set a limit for Co in drinking water. For centralized drinking-water sources, the cobalt limits are 50 μg/L for groundwater (GB/T 14848-2017, Class III) and 1.0 mg/L for surface water (GB 3838-2002). The measured Co2+ concentration in condensed water (0.0565 μg/L) equals 0.113% and 0.00565% of these two limits, respectively, confirming water safety.

Table S7. Environmental Risk Assessment (Based on Environmental Water Concentrations)

Metal	MEC (μg/L)	PNEC (μg/L)	Source	RQ	Risk level
Cu	0.875	1	Australia and New Zealand freshwater protection guideline[12]	0.875	Low risk ($0.1 \le RQ < 1$)
Ni	4.05	8	Australia and New Zealand freshwater protection guideline	0.506	Low risk ($0.1 \le RQ < 1$)
Co	11.3	4	US EPA chronic recommended value[13]	2.83	Potential risk (>1)

Note: Using the risk quotient (RQ) method: RQ = MEC / PNEC, where MEC is the maximum concentration in environmental water (336 h), and PNEC is the most stringent benchmark value for protecting aquatic organisms.

Table S8. Evaporation performance data under one sun irradiation

**Material**	**Total evaporation rate ($kg \cdot m^{-2} \cdot h^{-1}$)**	**Dark evaporation rate ($kg \cdot m^{-2} \cdot h^{-1}$)**	**Net evaporation rate ($kg \cdot m^{-2} \cdot h^{-1}$)**	**Corrected net efficiency**	Stable surface temperature T_1 (K)
Cu_7S_4-C	1.58	0.35	1.23	85.7%	326.35
Ni_3S_2-C	1.35	0.29	1.06	73.8%	325.45

Material	Total evaporation rate (kg·m^{-2}·h^{-1})	Dark evaporation rate (kg·m^{-2}·h^{-1})	Net evaporation rate (kg·m^{-2}·h^{-1})	Corrected net efficiency	Stable surface temperature T_1 (K)
Co_3S_4-C	1.47	0.32	1.15	80.1%	325.95
C-Amberlyst	1.21	0.27	0.94	65.2%	322.05
Lignocellulosic sponge	0.82	0.26	0.56	38.7%	318.25

Note: The total evaporation rate is the apparent evaporation rate under 1 Sun (Figure 11); the net evaporation rate is obtained by subtracting the dark evaporation rate from the apparent rate (Figure 9a); the corrected net efficiency is the interfacial evaporation efficiency calculated from the net evaporation rate (Figure 9a). T1 is the stable surface temperature under illumination (Figure 9b); ΔHLV and Q are calculated according to the equations in the main text. The gross (apparent) rates (Figure 11) and the net/dark rates (Figure 9a) were obtained in separate measurement series; small deviations from net = gross − dark arise from batch-to-batch variation.

Table S9. Synthesis parameters of the three composites derived from Amberlyst 15.

**Parameter**	**Cu_7S_4-C**	**Ni_3S_2-C**	**Co_3S_4-C**
Metal salt	$Cu(NO_3)_2 \cdot xH_2O$	$Ni(NO_3)_2 \cdot xH_2O$	$Co(NO_3)_2 \cdot xH_2O$
Metal salt : resin mass ratio	1 : 1	1 : 1	1 : 1
Impregnation time	8 h	8 h	8 h
Drying temperature / time	90 °C / 12 h	90 °C / 12 h	90 °C / 12 h
Carbonization temperature	800 °C	800 °C	800 °C
Carbonization time	2 h	2 h	2 h
Heating rate	~13 °C·min^{-1}	~13 °C·min^{-1}	~13 °C·min^{-1}
Atmosphere	Ar	Ar	Ar
Gas flow rate	50 mL·min^{-1}	50 mL·min^{-1}	50 mL·min^{-1}

References

[1] Guan HS, Fan TT, Bai HY, Su Y, Liu Z, Ning X, Yu M, Ramakrishna S, Long YZ (2022) A waste biomass-derived photothermic material with high salt-resistance for efficient solar evaporation. Carbon 188: 265-275. https://doi.org/10.1016/j.carbon.2021.12.029

[2] Song CY, Zhang BY, Hao L, Min JK, Liu N, Niu R, Gong J, Tang T (2022) Converting poly(ethylene terephthalate) waste into N-doped porous carbon as CO2

adsorbent and solar steam generator. Green Energy Environ 7: 411-422. https://doi.org/10.1016/j.gee.2020.10.002

[3] Guo HX, Li TJ, Ai S, He CY, Liu BH, Lu ZW, Wang WM, Liu MM, Wang XT, Liu G, Gao XH (2023) Evaporator fabricated with accessible photothermal material derived from waste fallen leaves for highly efficient desalination. Appl Surf Sci 619: 156728. https://doi.org/10.1016/j.apsusc.2023.156728

[4] Wang CF, Wu CL, Kuo SW, Hung WS, Lee KJ, Tsai HC, Chang CJ, Lai JY (2020) Preparation of efficient photothermal materials from waste coffee grounds for solar evaporation and water purification. Sci Rep 10: 12769. https://doi.org/10.1038/s41598-020-69778-2

[5]Li X, Li M, Shi Q, Guo H, Wang L, Guo X, Chen Z, Sessler JL, Xiao H, James TD (2022) Exhausted Cr(VI) Sensing/Removal Aerogels Are Recycled for Water Purification and Solar-Thermal Energy Generation. Small 18: 2201949. https://doi.org/10.1002/smll.202201949

[6]Zhao XZ, Liu CK (2020) Overcoming salt crystallization with ionic hydrogel for accelerating solar evaporation. Desalination 482: 114385. https://doi.org/10.1016/j.desal.2020.114385

[7]Qi ZQ, Zhang WB, Han XS, Yang KY, Cai HZ (2024) In-situ lignin regeneration facilitated corn straw-based photothermal evaporator with high cost-effectiveness. Ind Crop Prod 213: 118420. https://doi.org/10.1016/j.indcrop.2024.118420

[8]Li JY, Jing YJ, Zhou X, Mao JL, Chen YJ, Sun HX, Deng XF, Gao CZ (2021) Multifunctional photothermal materials based on natural pumices for high efficiency solar-driven interface evaporator. Int J Energy Res 45: 20132-20142. https://doi.org/10.1002/er.7090.

[9]State Administration for Market Regulation & Standardization Administration of China. Standards for Drinking Water Quality (GB 5749-2022); Standards Press of China: Beijing, China, 2022. Available online: https://openstd.samr.gov.cn/bzgk/std/newGbInfo?hcno=99E9C17E3547A3C0CE2FD1FFD9F2F7BE (accessed on 24 September 2026).

[10]State Administration for Market Regulation & Standardization Administration of China. Standard for Groundwater Quality (GB/T 14848-2017); Standards Press of China: Beijing, China, 2017. Available online: https://openstd.samr.gov.cn/bzgk/std/newGbInfo?hcno=F745E3023BD5B10B9FB5314E0FFB5523 (accessed on 24 September 2026).

[11]Ministry of Ecology and Environment of the People's Republic of China. Environmental Quality Standards for Surface Water (GB 3838-2002); China Environmental Science Press: Beijing, China, 2002. Available online: https://www.mee.gov.cn/ywgz/fgbz/bz/bzwb/shjbh/shjzlbz/200206/t20020601_66497.shtml (accessed on 24 September 2026).

[12]ANZG (Australian and New Zealand Guidelines). Australian and New Zealand Guidelines for Fresh and Marine Water Quality; Australian and New Zealand Governments and Australian state and territory governments: Canberra, ACT, Australia, 2018. Available online: https://www.waterquality.gov.au/anz-guidelines (accessed on 24 September 2026).

[13]U.S. Environmental Protection Agency. National Recommended Water Quality Criteria: Aquatic Life Criteria Table. Available online: https://www.epa.gov/wqc/national-recommended-water-quality-criteria-aquatic-life-criteria-table (accessed on 24 September 2026).
